# Distorted thoughts as a mediator of depressive symptoms in patients with major depressive disorder: a longitudinal study

**DOI:** 10.1186/s12955-023-02178-y

**Published:** 2023-08-14

**Authors:** Adriana Munhoz Carneiro, Danilo Assis Pereira, Fernando Fernandes, Makilim Nunes Baptista, André Russowsky Brunoni, Ricardo Alberto Moreno

**Affiliations:** 1 Mood Disorders Unit, Faculty of Medicine FMUSP, University of Sao Paulo, BR Ovideo Pires de Campos St., São Paulo, SP 785 05403-010 Brazil; 2 Independent Consultant, Brasilia, Brazil; 3 Laboratory of Psychometric Evaluation in Mental Health -LAPSAM III - São Francisco University, Campinas, Brazil; 4 Department of Internal Medicine, Faculty of Medicine FMUSP, University of Sao Paulo, São Paulo, SP Brazil

**Keywords:** Depressive disorder, Cognitive distortions, Thinking, Dysfunctional thinking, Depression, Mediation

## Abstract

**Background:**

Distorted thoughts are common in Major Depressive Disorder (MDD), and can impact patients’ perceptions of depression severity, and predict chronicity and treatment response. This study aimed to investigate whether distorted thoughts mediate depressive symptoms in MDD over a 6-month period.

**Method:**

These are secondary results from a study that followed 119 patients diagnosed with moderate to severe MDD for 6 months. Diagnoses were confirmed by the Structured Interview for DSM-IV (SCID-CV). The analysis was composed of results from the Hamilton Depression Rating Scale (HAMD-17), the Montgomery-Asberg Depression Rating Scale (MADRS), the second edition of the Beck Depression Inventory (BDI-II), and the Depression Thoughts Scale (DTS) collected at weeks 1, 8, 12 and 24.

**Results:**

Results showed that the DTS mediated the relationship between depressive symptoms experienced approximately 3 months after starting antidepressant treatment.

**Conclusion:**

Cognitive distortions were linked as a mediator to depressive symptoms, highlighting the importance of early psychological interventions in patients with MDD who exhibit these distortions.

**Trial registration:**

NCT02268487.

**Supplementary Information:**

The online version contains supplementary material available at 10.1186/s12955-023-02178-y.

Distorted thoughts, characterized by cognitive biases related to a pessimistic view about the patients themselves, the others/world, and their future are consistently associated with depression [1–3]. These thoughts almost always occur in depressed patients during the acute phase and are directly related to their severity [1, 3, 4]. Moreover, these thoughts not only persist in approximately 50% of remitted depression patients, but they are also predictors of depression chronicity [2, 5, 6].

One of the most comprehensive models to interpret the distorted thoughts in depression was proposed by Aaron Beck, the author of Cognitive Behavioral Therapy (CBT) [1]. According to the CBT model (the depression tripartite model), the presence of distorted thoughts increases in parallel with the severity of depression [1, 3]. Generally, those schemas co-occur, but one triad structure may work more negatively than others. For example, Wang et al. [6] found that in depressed patients, hopelessness (i.e., a negative view of the future) was the most important factor, whereas McIntosh and Fisher et al. [7] found that in a non-clinical sample, the most important factor was distorted self-centered thoughts.

While there is evidence linking distorted thoughts and depression, studies investigating the effects of how these thoughts affect people have yieded mixed results. Some studies have suggested negative affect as a predictor of depression severity [2, 8] and antidepressant treatment outcomes [9]. Lewinsohn [10] found that this dysfunctional way of thinking (measured by the DAS) was a predictor of future episodes. Şenormancı et al. [11] concluded that the level of excessive thinking mediated dysfunctional attitudes and depressive symptoms in MDD patients, supporting the idea that rumination works as a vulnerability factor for depression.

In contrast, Lewinnsohn et al. [10] observed that negative styles (i.e., the way they explain the events) and negative life events were predictive of MDD onset only when participants were under high levels of stress. Quigley et al. [12] found evidence for concurrent but not longitudinal associations between thoughts and symptom change, concluding that despite a relationship being found between thought distortions and symptom change, they were not longitudinal correlations (mediation effects), as theory postulates.

Although research suggests that thoughts are a significant feature of depression, much uncertainty still exists about their mechanism in patients with the disorder. Thus, this study investigated whether distorted thoughts mediate the depressive symptoms in patients with MDD, in order to gain a better understanding of the mechanism underlying this link. We hypothesize that distorted thoughts may have a moderate correlation with the severity and symptoms of depression, and we aim to investigate how this correlation is mediated.

## Methods

### Design

This is a secondary analysis from an open-label phase 3 study [13]. It was conducted in Sao Paulo, Brazil, between 2015 and 2019. All participants provided written informed consent, according to the AIUNI study protocol. During the study, patients received sertraline monotherapy for up to half a year, with doses ranging from 50 to 200 mg, adjusted to scores on the Hamilton Depression Scale (HAMD) [14] and the Udvalg for Kliniske Undersøgelser Side Effect Rating Scale (UKU; [15]). Further details of the AIUNI trial have been described previously [13].

### Participants

Patients were recruited online by responding to two screening questions about depressive symptoms: “Have you been feeling depressed for most of the day, on most days?” and “Have you been feeling less interested in or losing interest in things you normally like?” Those who answered “yes” to both questions received a phone screening from trained researchers who investigated depression and bipolar disorder symptoms using a checklist developed by the authors, adapted from DSM criteria [16]. Those who had significant depressive symptoms were scheduled for an in-person appointment with trained psychiatrists, who interviewed eligible patients in a pre-trial process. In sequence, they were transferred to a trained researcher who administered the Structured Clinical Interview for DSM-IV (SCID-CV) [17] and enrolled them in the study.

The eligibility criteria were: (1) age between 18–59 years; (2) MDD without comorbidities diagnosed by the Structured Clinical Interview for DSM-IV [17] a Hamilton Depression Scale (HAMD) [14] score ≥ 14 without psychotic symptoms; (3) without a past or present history of neurological disorders or severe head injury; (4) not being pregnant or with a severe/unstable medical condition, including cardiovascular, hepatic, endocrine, neurological, renal, agranulocytosis, or other clinically significant laboratory or electrocardiographic abnormalities; (6) requiring any psychotropic medication other than antidepressants (AD) except sedative or hypnotic to sleep; a severe risk of suicide (defined by a score of 3 in HAMD item 3); and (6) a score ≥ 2 in the Young Mania Rating Scale (YMRS) [18] Brazilian version [19]. We excluded all patients who stated that they were receiving another validated treatment (including psychotherapy) from the current study. We would like to highlight that our research took into consideration the SCID [17] combined with clinical diagnosis based on the current DSM manual for our inclusion criteria [16].

### Clinical measures

#### Depressive symptoms

The Brazilian version of the Hamilton Depression Rating Scale [20] (Fleck et al., 2004) and Montgomery-Asberg Depression Rating Scale -MADRS [21] translated by Dractu et al. [22] were used to evaluate depressive severity by trained clinicians. The self-assessment scales included were the Beck Depression Inventory—BDI-II [23] and the Depressive Thoughts Scale – DTS [24]. For BDI-II, we considered the guidelines for the Brazilian clinical version [25]. All cutoff points used to interpret results are available in Table S1, Supplementary material.

#### Depressive thoughts

The Depressive Thoughts Scale (DTS) [24] was developed based on the principles of the cognitive triad for depression proposed by Aaron T. Beck [3]. The DTS is a three-point Likert scale and has 27 items, divided into two factors: F1 (low self-esteem/hopelessness) with 16 items, referring to items about thoughts of defeat, negative view of the future and life and F2 (interpersonal relationship) with 11 items reflecting thoughts of feeling supported, understood, and cared for. The analyses of internal structure using principal component analysis indicated a two-factor solution with an explained total variance of 49.74%. For the model used in the present study, i.e., 27-item, the reliability indices were *α* = 0.93 for F1, *α* = 0.89 for F2, and the total scale had a reliability of *α* = 0.93, confirming that overall, the scale had an excellent reliability rating [24]. In this version, the items and eigenvalues for each factor were as follows: F1 (low self-esteem/hopelessness) with 16 items (36.82% of variance).

### Procedures

Ethical approval was obtained from the University of São Paulo ethics committee (CAE 02222012.8.0000.0068) in accordance with the Declaration of Helsinki. Patients were recruited from a tertiary outpatient Mood Disorders Unit, diagnosed according to the current DSM manual version [16], and included in the study after they had read, understood, and signed the informed consent form, according to the AIUNI protocol study [13]. The assessment took an average of 30 min and was applied at weeks one (baseline), four, eight, twelve, and twenty-four – (Weeks (W) 1, 8, 12, and 24).

The study had a high dropout rate of 27% up to W8, 38% up to W12, and 69% up to W24. The main reasons for dropouts were the following: monotherapy (drug-related factor), patient-related variables (economic variable), and physician-related factors (communication and a lack of psychoeducation). Some patients stated that they are already feeling well and no longer require physician appointments, while others stated that they have returned to work and no longer have time to participate in the study. Considering the number of dropouts, we performed an analysis to compare study completers to non-completers. These findings showed no statistical significance (*p* > 0.05), indicating that patients who did not complete the trial had similar depression and dysfunctional thoughts as those who did. (Table S2, supplementary material).

## Statistical analyses

The analysis of variance of repeated measures (ANOVArm) was used to compare mean differences longitudinally. By default, rmANOVA makes the following assumptions: a) for each group, the dependent variable is normally distributed; b) the covariate and the experiment effect are independent; c) the sphericity assumption is satisfied. Sphericity indicates that the repeated measures’ differences’ variances are all equal. The confidence interval for the mean difference was calculated with post hoc tests. The magnitude of the observed effect is shown. The effect size is calculated using Cohen’s *d*. We avoided Type I errors by using the Holm method.

For the mediation analysis, the only request was that the DTS F1/F2, considered an independent variable (IV), occur before the DTS F1/F2 mediation variable and, consequently, before the depressive symptoms measured by the HAMD, MADRS, and BDI-II, considered a dependent variable (VD). We conducted all analyses using the four assessment moments described: First assessment (Week-): baseline occurred approximately 1 week before patients initiated antidepressant treatment. The HAM-D, MADRS, DTS and BDI-II were administered to measure the presence and severity of symptoms and depressive thoughts. Second assessment (Week-4) occurred approximately 4 weeks after patients initiated antidepressant treatment. As in time 1, HAM-D, MADRS, DTS and BDI-II were administered to measure the presence and severity of symptoms and depressive thoughts. Third assessment (Week-12): occurred approximately 3 months after the beginning of antidepressant treatment. The same scales were applied. Forth assessment (Week-24): occurred approximately 6 months after the antidepressant treatment, and the same scales were applied.

## Results

### Characteristics of the sample

**Fig. 1:**
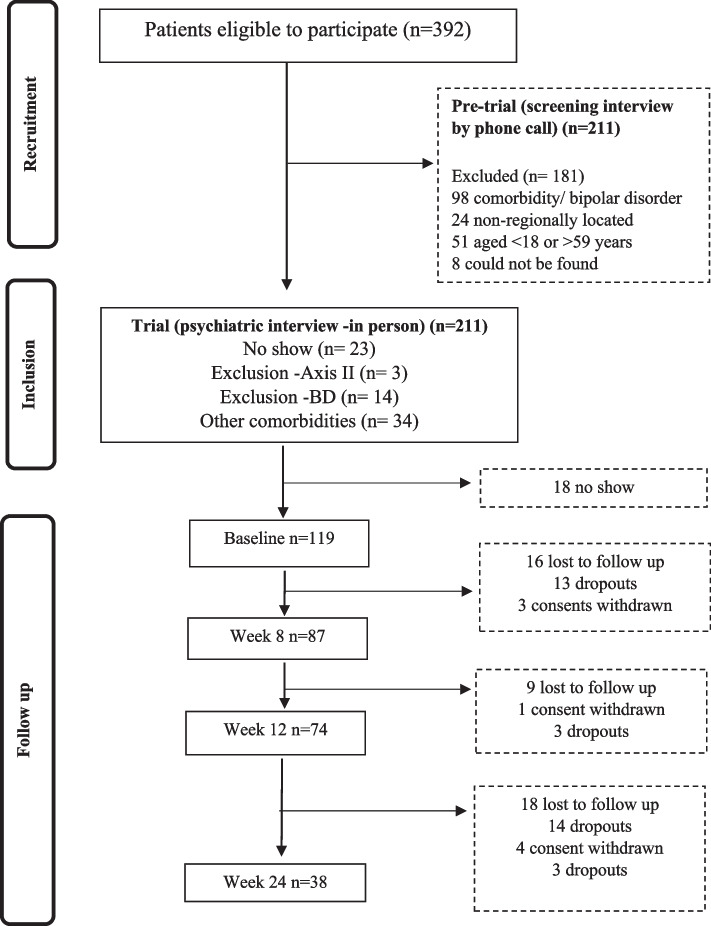
Flow diagram of study participants

One hundred nineteen patients were considered in our analysis, with a mean age of 40.6 years (SD = 11). Most participants were female (*n* = 85, 72%), and the sample had an average of 14 formal years of education (SD = 4.8). All the participants were diagnosed with MDD with melancholic features. Most participants (*n* = 79, 66.38%) had a recurrent episode, 32.77% (*n* = 39) indicated that this was their second episode, and only one was in their first episode. Patients were followed weekly, and 38 (31%) patients finished the study (for more information, see the flow diagram presented in Fig. 1).

The means (M) and standard deviations (SD) for each test used in weeks 1, 8, 12, and 24 are shown in Table 1. It can be seen in all measurements that, on average, patients responded positively to pharmacological treatment.

**Table 1:** Changes in depression severity (symptomatology and thoughts) over time for groups that finished the study (*n* = 38) during the 6-month trial

**Test**	**Week 1**	**Week 8**	**Week 12**	**Week 24**	**F**	***p*-value**	**η**^**2**^
HAMD	19.7 (4.5)	6.8 (4.0)	7.9 (5.0)	4.8 (4.6)	100.35	< .001	.73
MADRS	25.4 (6.9)	11.0 (7.6)	9.1 (7.5)	6.1 (6.7)	68.87	< .001	.66
BDI-II	35.8 (7.0)	14.1 (10.1)	14.1 (8.9)	11.6 (9.2)	68.05	< .001	.76
DTS-F1	36.3 (6.3)	25.1 (7.5)	26.2 (8.7)	26.0 (8.3)	25.36	< .001	.56
DTS-F2	21.4 (4.3)	17.2 (4.1)	16.5 (5.3)	16.9 (5.5)	10.94	< .001	.33

Repeated measures ANOVA within-subjects effects. Type III partial sum of squares was selected because it takes interactions into account. Here, the repeated measures factors of interest and their associated levels can be labeled. η^2^ = eta-squared as an estimate of the effect size

*HAMD* Hamilton Scale of Depression, *MADRS* Montgomery Asberg depression scale, *BDI-II* Beck Depression Scale 2nd Edition, *DTS* Depressive Thoughts Scale, *DTS-F1* Factor 1 (low self-esteem/ hopelessness), *DTS-F2* factor 2 (interpersonal relationship)

Moreover, analyses of repeated measures (ANOVArm) were used to distinguish statistically significant results between assessment periods, showing an important reduction in depressive symptoms as well as distorted thoughts (Table 1). Complementary analysis of the ANOVArm (available at Supplementary material -Table S3) revealed differences between week-1 and all subsequent weeks (*p* = 0.001), but not between weeks 8 and 12. Regarding DTS results, between weeks 1 and 8, effect sizes (Cohen’s d) were very large in F1 (> 1.30) and for DTS F2 of the DTS, the Cohen’s d ranged between 0.89 and 1.04 (large effect).

In sum, these results suggest that both F1 and F2 scores of the DTS scale significantly decreased from Week 1 to Week 8, with no significant further change thereafter up to Week 24. The large effect sizes indicate a strong practical significance in these reductions from W1 to W8. The lack of significant differences post Week 8 suggests that the intervention’s effects might have stabilized by this time point. Once results showed that patients recovered from their major depressive symptoms but not their thoughts, we decided to perform a mediation analysis to better understand this relationship.

### Mediation of dysfunctional thoughts and depressive symptoms

In the second set of results, we performed a mediation analysis. We analyzed all possible time combinations for the first and second factors (F1 and F2) of the Dysfunctional Thoughts Scale (DTS), with the aim of identifying if DTS could potentially mediate the outcomes of the depressive scales—in other words, depressive symptoms (Table 2 shows the more relevant results from this analysis).

**Table 2:** Summary of main mediation results

**Model**		**0- > 8- > 12**		**0- > 8- > 24**		**0- > 12- > 24**	
**IV- > MV- > DV**		**R2** **(%)**	**CI** **LL;UL**	**R2** **(%)**	**CI** **LL;UL**	**R2** **(%)**	**CI** **LL;UL**
F1							
F1	HAMD	24.0^***^	.07;.31	40.7^***^	-.14;.26	19.3^**^	-.02;.14
F1	MADRS	25.4^***^	.12;.52	38.7^***^	-.28;.30	20.5^**^	-.08;.17
F1	BDI-II	28.0^***^	.22;.77	32.5^**^	.08;.83	21.5^*^	.03;.63
F2	BDI-II	8.9	.04;.33	37.6^**^	.25;.85	28.5^**^	.02;.70
F2							
F2	HAMD	33.8^***^	.10;.61	30.7^***^	-.05;.35	17.8^**^	-0.06;.27
F2	MADRS	34.0^***^	.20;.98	32.3^***^	-.06;.64	18.2^**^	-.08;.42
F2	BDI-II	33.9^***^	.45;1.39	48.3^***^	.51;1.56	36.2^**^	.16;1.32
F1	BDI-II	3.9	-.09;.8	18.0^**^	.05;1.03	6.9	-.06;.69

*F1* DTS Scale Factor 1 (low self-esteem/ hopelessness), *F2* DTS Scale Factor 2 (interpersonal relationship), *HAMD* Hamilton Depression Scale, *MADRS* Montgomery-Äsberg Depression Rating Scale, *BDI-II* Beck Depression Inventory, *LL* Lower limit (2.5% CI), *UL* Upper limit (97.5% CI)

< 0.1; ^*^ < 0.05; ^**^ < 0.01; ^***^ < 0.001

Table 2 encompasses the models, Independent Variables (IV), Mediating Variables (MV), and Dependent Variables (DV) along with their corresponding R-squared values (R2). Please take note of the levels of significance (*p*-values), depicted as follows: < 0.1; * < 0.05; ** < 0.01; *** < 0.001. In the table, F1 and F2 refer to the DTS Scale Factors 1 (low self-esteem/hopelessness) and 2 (interpersonal relationship) respectively. HAMD, MADRS, and BDI-II denote the Hamilton Depression Scale, Montgomery-Äsberg Depression Rating Scale, and 2nd Edition of the Beck Depression Inventory, respectively.

In the context of the correlation between DTS F1 and the Hamilton Depression Scale (HAMD) as a dependent variable at Week 12, it was found that both F1 and F2 mediated the relationship between DTS and HAMD, which indicates that both F1 and F2 can be considered as mediators for HAMD responses during Week 12. Comparable findings were observed when considering the Montgomery-Äsberg Depression Rating Scale (MADRS) as a dependent variable at Week 12. Both F1 and F2 mediated the relationship between DTS and MADRS.

When analyzing the BDI-II as a dependent variable, three key mediations were identified: Firstly, at Week 12, both F1 and F2 mediated the relationship between DTS and BDI-II. Secondly, at Week 8, F2 was observed to mediate the relationship between F1 at baseline and BDI-II at Week 12. Lastly, at Week 24, both F1 and F2 were found to mediate the relationship between DTS and BDI-II.

In addition, the roles of F2 and F1 at Week 8 were explored as mediators for the relationship between F1/F2 at baseline and BDI-II at Week 24. Finally, when analyzing BDI-II as a dependent variable at Week 24, it was observed that F1 and F2 mediated the relationship between DTS and BDI-II. It was also found that DTS F2 at Week 12 mediated the relationship between F1 at baseline and BDI-II at Week 24.

## Discussion

This study investigated whether dysfunctional thoughts mediated depressive symptoms. The study found that dysfunctional thoughts were not only correlated with depressive symptoms but also, moderated some results. The major finding indicated that the perception of self and future (F1) and the perception of the world and others (F2) in W8 mediated the relationships between the perception of depression symptoms (BDI-II) and severity of depression symptoms (MADRS and HAMD) in W12, i.e., those with dysfunctional interpretations of themselves, the world, and others in W8 have a more negative symptomatology response in W12.

This way, patients who had more dysfunctional interpretations of themselves, the world, and others in the second month of treatment tended to have a more negative symptomatologic response in the fourth month. These findings support the tripartite model proposed by Beck, indicating that thoughts can mediate depression severity [1, 3, 11]. Şenormancı et al. [11] reported comparable results in a similar sample to ours, reporting that rumination was a vulnerability factor for depression in those patients. Additionally, findings support the idea that negative affect could be a predictor of depression severity [8] and antidepressant treatment outcome [9].

Since these results differ from the longitudinal study by Quigley et al. [12], which found evidence for concurrent but not longitudinal associations between cognitive and symptom change, we hypothesized that measuring dysfunctional thoughts by the EPD, a more specific measure for distorted thoughts in depression, may have contributed to these differences. In addition, it is interesting to note that participants in the Quingley et al. [12] study received not only pharmacological treatment but also CBT.

Although depressive symptoms were in remission by week 8 and patients were characterized as having “limited” distortions by the DTS manual at week 12, their scores when compared to a non-clinical group are labeled as “very distorted,” i.e., they continue to display important dysfunctional thinking when compared to healthy controls. Results were different from Quigley et al. [12] study, which found no mediation between distorted thoughts and depressive symptoms but corroborated other findings indicating that dysfunctional thoughts continue even after the reduction of depressive symptoms [2, 5, 6].

Neither the perception about themselves or the future (DTS-F1) nor their relationships (DTS- F2) in 2 months mediated the depression severity measured by the HAMD or MADRS scales at the end of the 6 months. On the other side, for BDI-II, results indicated that dysfunctional thoughts mediate the severity of depression during the treatment. An important implication of the current findings is related to the scales; once symptoms have been operationalized, they vary considerably.

The differences between DTS and the rating scales (HAMD and MADRS) were expected, considering that only two HAMD items (feelings of guilt and suicide) and two MADRS items (pessimistic thoughts and suicidal thoughts) can be considered relevant to cognitive distortions. On the other hand, the BDI-II contains eight relevant items, i.e., 1/3 of the inventory items (pessimism, past failure, guilty feelings, punishment feelings, self-dislike, self-criticalness, suicidal thoughts or wishes, and worthlessness), and was developed by Beck, which could explain the mediation effects.

As symptoms decrease, thoughts become more prominent, demonstrating the distinction between symptomatic remission and full recovery, an important aspect when treating depression, as discussed in previous studies [11, 12]. It is hypothesized that future treatments should identify individuals at considerable risk and detect and prevent depression through early intervention in the thought domain. Additional research should be conducted to describe and control characteristics such as the number of episodes, age, severity, and age of onset [4, 26].

In fact, negative self- and future perceptions have been proven to be linked with depressive symptoms. This finding is consistent with Wang et. [6] study that found hopelessness (i.e., a negative view about the future) as a central point and with McIntosh and Fisher [7] study, which found that the main cognitive triad factors in a non-clinical group were distorted thoughts related to themselves. On the other hand, our findings also showed that the “functional relationship” dimension from DTS (i.e., the perception of being supported by others and having someone reliable) mediated the relation between dysfunctional thoughts about themselves and the future at the end of the second, fourth, and sixth months, indicating that the central point of distorted thoughts in our population was related to the negative view of others and the world. The perception of being supported, by someone with whom you can share your vulnerabilities is in line with the findings of Lewinsohn [10, 27] studies, which indicated that life stress (external events) could function as a stronger MDD predictor than dysfunctional thinking.

Research has already found that cognitive distortions persist not only in remitted people but are also considered predictors of depression chronicity [2, 4, 28]. These findings raise the possibility that treatments targeting specifically dysfunctional thoughts after treatment (as booster treatment) could potentially help to prevent recurrent depression rates and increase response to treatment, as proposed by other authors [26, 28].

This study has strengths and weaknesses. Regarding strengths, this is one of the first studies to use a specific scale based on Beck’s Cognitive Triad Theory with a sample of MDD patients without comorbidities over a 6-month period. The study also has important implications for depression, suggesting that future treatments should look for cognitive distortions as an important feature for early detection of MDD. Once results show that when AD is used to treat depression, distorted thoughts do get less, but not as much as was needed, maybe distorted thinking responses should be viewed in a new light: as more than a state, as a trait.

The current study had limitations, including the small sample and the sample dropout rates, which were significant (around 80%). Although those rates are not expected in North America or Europe longitudinal studies (around 30-40%), they could be found in other recently published Brazilian studies. As discussed, one of the highest reasons for poor adherence was related to a drug-related factor (tolerance, and monotherapy), followed by patient-related variables (economic status) and physician-related factors (communication and lack of psychoeducation to promote treatment adherence).

Another limitation was the absence of control groups, which would have allowed us to determine if another treatment, such as CBT or psychoeducation, could impact a better DTS score response and adherence to the study. To keep the study as close to understanding and evaluating the assumptions of the cognitive model as possible, we excluded patients undergoing psychotherapy, but one of the possible reasons for dropout could be attributed to a lack of psychoeducation, a variable that we did not control.

## Conclusions

Our findings contribute to the literature by generating new information regarding the mediation role of depressive thoughts on depression. It was found that the presence of cognitive distortions was linked to depressive symptoms, and this relationship was partially explained by the DTS scale. Future research should investigate whether cognitive distortions can serve as a predictive marker to identify which patients are more likely to respond favorably to specific types of treatment (e.g., pharmacological, neuromodulation, or psychological treatment) in order to prevent recurrences and control polymorphisms. Early detection and treatment of depressive symptoms are crucial for preventing depression. Follow-up studies to identify factors that can contribute to a higher risk of recurrence than those with lower levels of distortion are recommended. We believe that studies comparing euthymic and symptomatic states in the same patient can also provide valuable insights in this field. Overall, we conclude that incorporating a cognitive distortion evaluation into the treatment plan may be advantageous in achieving better treatment outcomes in MDD.

### Supplementary Information

**Additional file 1: Table S1.** Cutoff points used to categorize patients (levels) according to the results of the tests. **Table S2.** Mean differences between patients at baseline. **Table S3.** Post hoc comparisons in depressive symptomatology (HAMD, MADRS, BDI-II) and depressive thoughts (DTS) between weeks. **Figure S1.** Analysis of variance of repeated measures (ANOVArm) to compare mean differences longitudinally.

## Data Availability

The datasets used and analyzed during the current study are available from the corresponding author on reasonable request.

## Abbreviations

MDD: Major depressive disorder
AIUNI: Integral Assessment in Unipolar Depression
HAMD: Hamilton Scale of Depression
MADRS: Montgomery Asberg depression scale
BDI-II: Beck Depression Scale 2nd Edition
DTS: Depressive Thoughts Scale
DTS-F1: Factor 1 (low self-esteem/ hopelessness)
DTS-F2: Factor 2 (interpersonal relationship)

## References

[1] Beck AT, Bredemeier K (2016). A unified model of depression: integrating clinical, cognitive, biological, and evolutionary perspectives. Clin Psychol Sci.

[2] Timm C, Ubl B, Zamoscik V, Ebner-Priemer U, Reinhard I, Huffziger S (2017). Cognitive and affective trait and state factors influencing the long-term symptom course in remitted depressed patients. PLoS One.

[3] Beck AT. Cognitive therapy of depression. Guilford Press; 1979. p. 425.

[4] Conradi HJ, Ormel J, de Jonge P (2011). Presence of individual (residual) symptoms during depressive episodes and periods of remission: a 3-year prospective study. Psychol Med.

[5] Chahar Mahali S, Beshai S, Feeney JR, Mishra S (2020). Associations of negative cognitions, emotional regulation, and depression symptoms across four continents: International support for the cognitive model of depression. BMC Psychiatry.

[6] Wang L, Liu L, Shi S, Gao J, Liu Y, Li Y (2013). Cognitive trio: relationship with major depression and clinical predictors in Han Chinese women. Psychol Med.

[7] McIntosh CN, Fischer DG (2000). Beck’s cognitive triad: one versus three factors. Can J Behav Sci.

[8] Vrieze E, Demyttenaere K, Bruffaerts R, Hermans D, Pizzagalli DA, Sienaert P (2014). Dimensions in major depressive disorder and their relevance for treatment outcome. J Affect Disord.

[9] Uher R, Perlis RH, Henigsberg N, Zobel A, Rietschel M, Mors O (2012). Depression symptom dimensions as predictors of antidepressant treatment outcome: replicable evidence for interest-activity symptoms. Psychol Med.

[10] Lewinsohn PM, Joiner TE, Rohde P (2001). Evaluation of cognitive diathesis-stress models in predicting major depressive disorder in adolescents. J Abnorm Psychol.

[11] Şenormancı Ö, Yılmaz AE, Saraçlı Ö, Atasoy N, Şenormancı G, Atik L (2014). The mediator role of ruminative thinking style in the relationship between dysfunctional attitudes and depression. Compr Psychiatry.

[12] Quigley L, Dozois DJA, Bagby RM, Lobo DSS, Ravindran L, Quilty LC (2019). Cognitive change in cognitive-behavioural therapy pharmacotherapy for adult depression: a longitudinal mediation analysis. Psychol Med.

[13] Fernandes FF, Moreno RA. Integral assessment in Unipolar Depression (AIUNI). Clinical Trials.gov, NCT02268487. 2014.

[14] Hamilton M. A rating scale for depression. 1960. p. 7. 10.1136/jnnp.23.1.56 PMC495331 14399272

[15] Lingjaerde O, Ahlfors UG, Bech P, Dencker SJ, Elgen K (1987). The UKU side effect rating scale. A new comprehensive rating scale for psychotropic drugs and a cross-sectional study of side effects in neuroleptic-treated patients. Acta Psychiatr Scand Suppl.

[16] American Psychiatric Association [APA]. Diagnostic and Statistical Manual of Mental Disorders, 5th ed, Text Revision (DSM-5). Washington, DC; 2013.

[17] First MB, Spitzer RL, Gibbon M, Williams JBW (1995). The Structured Clinical Interview for DSM-III-R Personality Disorders (SCID-II). Part I: Description. J Pers Disord.

[18] Young RC, Biggs JT, Ziegler VE, Meyer DA (1978). A rating scale for mania: reliability, validity and sensitivity. Br J Psychiatry.

[19] Vilela JAA. Estudo da confiabilidade e validade de uma versão modificada da Young Mania Rating Scale. Masters Dissertation: Faculdade de Medicina de Ribeirão Preto; 2000.

[20] de Almeida Fleck MP, Chaves ML, Poirier-Littré MF, Bourdel MC, Loo H, Guelfi JD (2004). Depression in France and Brazil: factorial structure of the 17-item Hamilton depression scale in inpatients. J Nerv Ment Dis.

[21] Montgomery SA, Asberg M (1979). A new depression scale designed to be sensitive to change. Br J Psychiatry.

[22] Dratcu L, da Costa Ribeiro L, Calil HM (1987). Depression assessment in Brazil. The first application of the Montgomery-Asberg Depression Rating Scale. Br J Psychiatry.

[23] Beck AT, Steer RA, Brown G. Beck depression inventory–II. PsycTESTS Dataset. 2011. 10.1037/t00742-000.

[24] Carneiro AM, Baptista MN (2016). Escala de Pensamentos Depressivos- EPD. Manual.

[25] Gorenstein C, Pang WY, Argimon IL, Werlang BSG (2011). Inventário Beck de Depressão-II. Manual.

[26] Guidi J, Fava GA (2021). Sequential combination of pharmacotherapy and psychotherapy in major depressive disorder: a systematic review and meta-analysis. JAMA Psychiat.

[27] Lewinsohn PM, Allen NB, Seeley JR, Gotlib IH (1999). First onset versus recurrence of depression: differential processes of psychosocial risk. J Abnorm Psychol.

[28] DeRubeis RJ, Zajecka J, Shelton RC, Amsterdam JD, Fawcett J, Xu C (2020). Prevention of recurrence after recovery from a major depressive episode with antidepressant medication alone or in combination with cognitive behavioral therapy: phase 2 of a 2-phase randomized clinical trial. JAMA Psychiat.

