# Supplementary material for: Distorted thoughts as a mediator of depressive symptoms in patients with major depressive disorder: a longitudinal study
Source: Health Qual Life Outcomes. 2023 Aug 14;21:88. doi: 10.1186/s12955-023-02178-y (PMC10424419; doi:10.1186/s12955-023-02178-y)
Supplement: Supplementary file 1 — Additional file 1: Table S1. Cutoff points used to categorize patients (levels) according to the results of the tests. Table S2. Mean differences between patients at baseline. Table S3. Post hoc comparisons in depressive symptomatology (HAMD, MADRS, BDI-II) and depressive thoughts (DTS) between weeks. Figure S1. Analysis of variance of repeated measures (ANOVArm) to compare mean differences longitudinally. [file 12955_2023_2178_MOESM1_ESM.docx]

**Supplementary material**

**Table S1.** Cutoff points used to categorize patients (levels) according to the results of the tests.

| **Level** | **HAMD** | **MADRS** | **BDI-II** | **DTS-F1** | **DTS-F2** |
| --- | --- | --- | --- | --- | --- |
| Regular | < 8 | < 9 | < 10 | < 27 | < 18 |
| Low/Mild | 8-13 | 9-17 | 10-18 | 27-32 | 18-22 |
| Moderate | 14-18 | 18-34 | 19-29 | 33-41 | 23-26 |
| Severe | > 18 | > 34 | 30-63 | > 41 | > 26 |

***Note:*** *HAMD = Hamilton Scale of Depression; MADRS = Montgomery Asberg depression scale; BDI-II = Beck Depression Scale 2nd Edition; DTS = Depressive Thoughts Scale; DTS-F1 = factor 1 (low self-esteem/ hopelessness); DTS-F2 = factor 2 (interpersonal relationship).*

**Table S2.** Mean differences between patients at baseline.

|  | **Group** | **Mean** | **SD** | **t [df]** | ***p*** |
| --- | --- | --- | --- | --- | --- |
| HAMD | 1 | 19.56 | 5.16 | 0.81 [39.60] | *0.43* |
|  | 2 | 18.71 | 4.50 |  |  |
| MADRS | 1 | 25.34 | 10.12 | 0.20 [49.83] | *0.85* |
|  | 2 | 25.00 | 7.02 |  |  |
| YMRS | 1 | 1.86 | 2.57 | -0.50 [39.46] | *0.63* |
|  | 2 | 2.12 | 2.25 |  |  |
| BDI | 1 | 31.30 | 12.20 | -2.02 [51.21] | *0.49* |
|  | 2 | 35.54 | 8.27 |  |  |
| DTS F1 | 1 | 35.79 | 7.00 | -0.35 [35.01] | *0.73* |
|  | 2 | 36.33 | 7.12 |  |  |
| DTS F2 | 1 | 19.94 | 4.71 | -1.32 [34.32] | *0.20* |
|  | 2 | 21.41 | 4.94 |  |  |

***Note****: Group 1 = patients who did not complete the study; Group 2 = patients who completed the study.*

***Note 2:*** *HAMD = Hamilton Scale of Depression; MADRS = Montgomery Asberg depression scale; BDI-II = Beck Depression Scale 2nd Edition; DTS = Depressive Thoughts Scale; DTS-F1 = factor 1 (low self-esteem/ hopelessness); DTS-F2 = factor 2 (interpersonal relationship).*

Considering the results by the cutoff points, it was noticed that patients did not achieve remission in the BDI-II (< 10 points), in contrast to results found in HAMD (< 8) or MADRS (< 9). In DTS, patients initially reported a moderate level (M=36.3) of low self-esteem and hopelessness score (DTS-F1), and, from the eighth week, they reported minimal cognitive distortions (M=25.1). For DTS-F2, results indicated that patients initially did not perceive relationship difficulties (M=21.4), but a significant improvement was observed at week 8 (M=17.2), indicating that patients began to re-interpret their relationships.

**Table S3** - Post hoc comparisons in depressive symptomatology (HAMD, MADRS, BDI-II) and depressive thoughts (DTS) between weeks.

| **Post hoc comparisons** | | **Mean difference** | **Lower** | **Upper** | **t** | **Cohen's d** | **p_holm_** |
| --- | --- | --- | --- | --- | --- | --- | --- |
| HAMD 1 | HAMD 8 | 12.92 | 10.34 | 15.51 | 13.42 | 2.18 | < .001 |
|  | HAMD 12 | 12.55 | 9.97 | 15.14 | 13.04 | 2.16 | < .001 |
|  | HAMD 24 | 14.95 | 12.36 | 17.53 | 15.53 | 2.52 | < .001 |
| HAMD 8 | HAMD 12 | -0.37 | -2.95 | 2.22 | -0.38 | -0.06 | 0.703 |
|  | HAMD 24 | 2.03 | -0.56 | 4.61 | 2.11 | 0.34 | 0.075 |
| HAMD 12 | HAMD 24 | 2.40 | -0.19 | 4.98 | 2.49 | 0.40 | 0.043 |
|  |  |  |  |  |  |  |  |
| MADRS 1 | MADRS 8 | 14.39 | 10.46 | 18.32 | 9.85 | 1.64 | < .001 |
|  | MADRS 12 | 16.36 | 12.43 | 20.29 | 11.19 | 1.87 | < .001 |
|  | MADRS 24 | 19.28 | 15.35 | 23.21 | 13.19 | 2.20 | < .001 |
| MADRS 8 | MADRS 12 | 1.97 | -1.96 | 5.90 | 1.35 | 0.23 | 0.180 |
|  | MADRS 24 | 4.89 | 0.96 | 8.82 | 3.35 | 0.56 | 0.003 |
| MADRS 12 | MADRS 24 | 2.92 | -1.01 | 6.85 | 2.00 | 0.33 | 0.097 |
|  |  |  |  |  |  |  |  |
| BDI 1 | BDI 8 | 21.77 | 16.42 | 27.07 | 11.19 | 2.39 | < .001 |
|  | BDI 12 | 21.68 | 16.38 | 26.98 | 11.14 | 2.38 | < .001 |
|  | BDI 24 | 24.27 | 18.97 | 29.57 | 12.47 | 2.66 | < .001 |
| BDI 8 | BDI 12 | -0.09 | -5.39 | 5.21 | -0.05 | -0.01 | 0.963 |
|  | BDI 24 | 2.50 | -2.80 | 7.80 | 1.29 | 0.27 | 0.564 |
| BDI 12 | BDI 24 | 2.59 | -2.71 | 7.89 | 1.33 | 0.28 | 0.564 |
|  |  |  |  |  |  |  |  |
| DTS-F1 1 | DTS-F1 8 | 11.14 | 7.10 | 15.19 | 7.51 | 1.64 | < .001 |
|  | DTS-F1 12 | 10.10 | 6.05 | 14.14 | 6.81 | 1.49 | < .001 |
|  | DTS-F1 24 | 10.33 | 6.29 | 14.38 | 6.97 | 1.52 | < .001 |
| DTS-F1 8 | DTS-F1 12 | -1.05 | -5.09 | 3.00 | -0.71 | -0.15 | 1.000 |
|  | DTS-F1 24 | -0.81 | -4.86 | 3.24 | -0.55 | -0.12 | 1.000 |
| DTS-F1 12 | DTS-F1 24 | 0.24 | -3.81 | 4.29 | 0.16 | 0.04 | 1.000 |
|  |  |  |  |  |  |  |  |
| DTS-F2 1 | DTS-F2 8 | 4.13 | 1.51 | 6.76 | 4.28 | 0.89 | < .001 |
|  | DTS-F2 12 | 4.83 | 2.20 | 7.45 | 5.00 | 1.04 | < .001 |
|  | DTS-F2 24 | 4.48 | 1.85 | 7.10 | 4.64 | 0.97 | < .001 |
| DTS-F2 8 | DTS-F2 12 | 0.70 | -1.93 | 3.32 | 0.72 | 0.15 | 1.000 |
|  | DTS-F2 24 | 0.35 | -2.28 | 2.97 | 0.36 | 0.08 | 1.000 |
| DTS-F2 12 | DTS-F2 24 | -0.35 | -2.97 | 2.28 | -0.36 | -0.08 | 1.000 |

***Note****. Post hoc comparisons = the first two columns correspond to the repeated measures factor levels compared. Mean difference = the mean difference between the levels. The 95% confidence interval (CI) of the mean difference between the compared levels. Lower = the lower bound of the CI. Upper = the upper bound of the CI. t = the value of the t-statistic. Cohen's d = the effect size Cohen's d. Cohen's d does not correct for multiple comparisons. p-holm = Holm's corrected p-value for multiple comparisons.*

***Note 2:*** *HAMD = Hamilton Scale of Depression; MADRS = Montgomery Asberg depression scale; BDI-II = Beck Depression Scale 2nd Edition; DTS = Depressive Thoughts Scale; DTS-F1 = factor 1 (low self-esteem/ hopelessness); DTS-F2 = factor 2 (interpersonal relationship).*


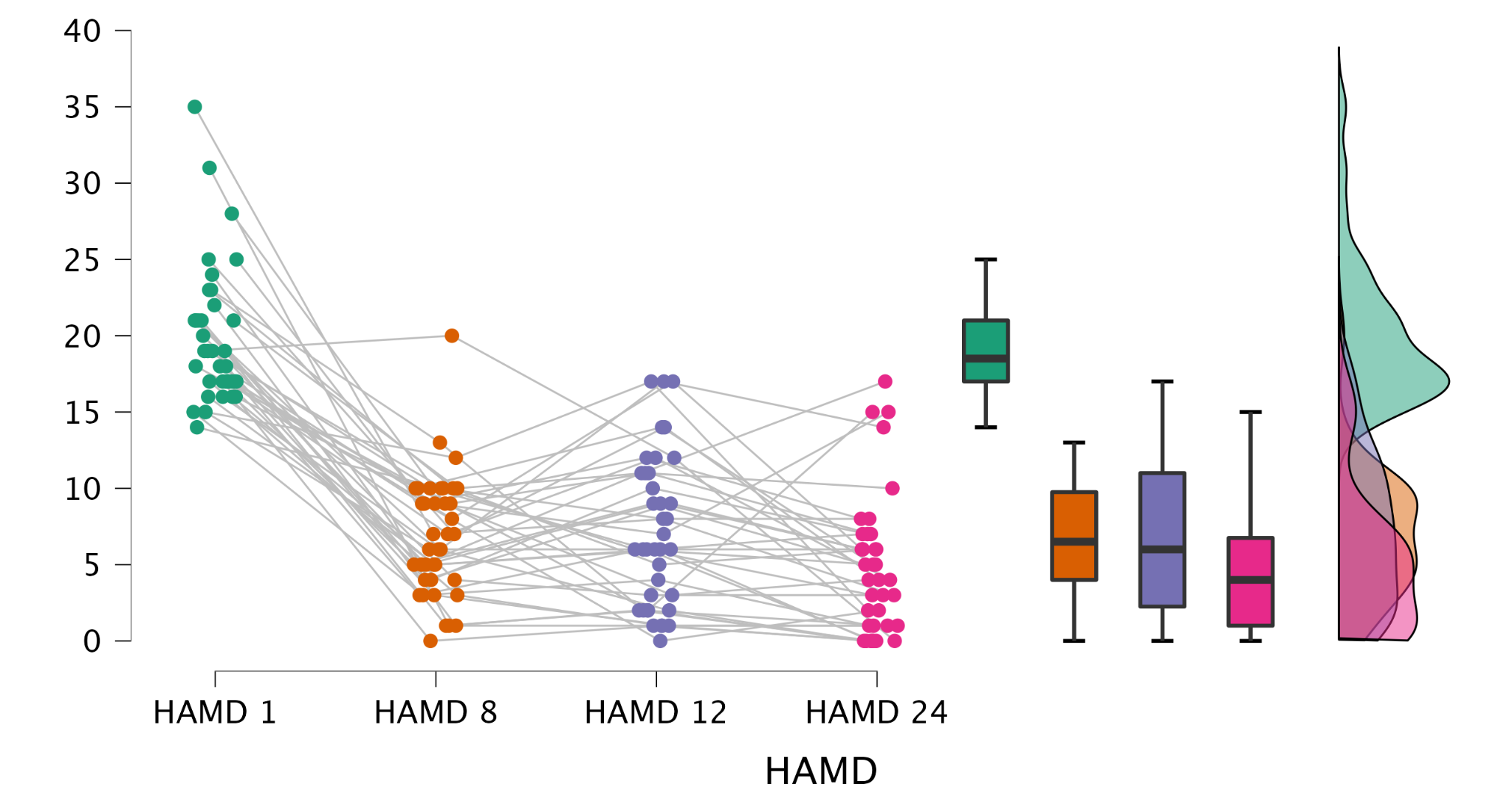

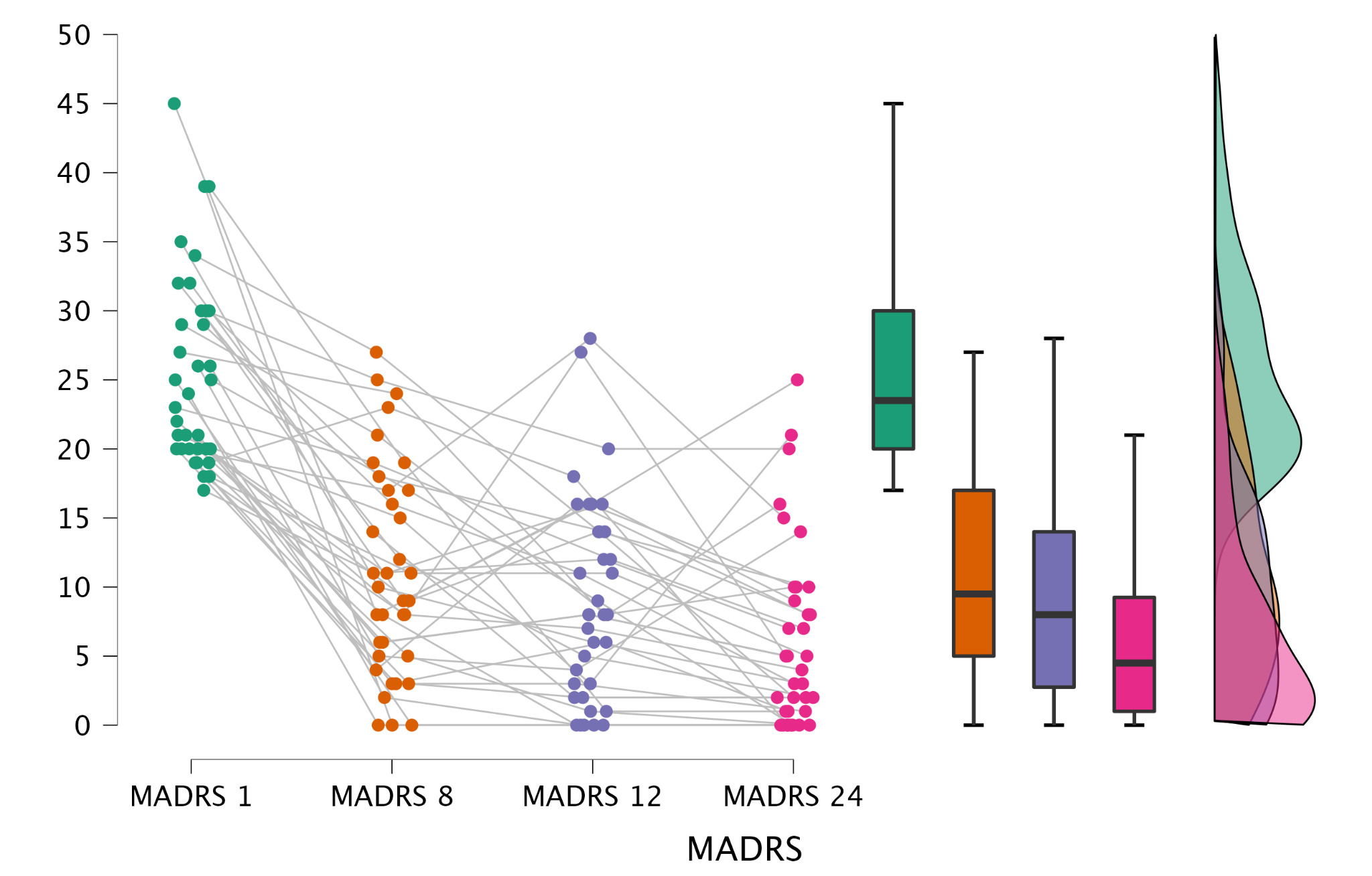


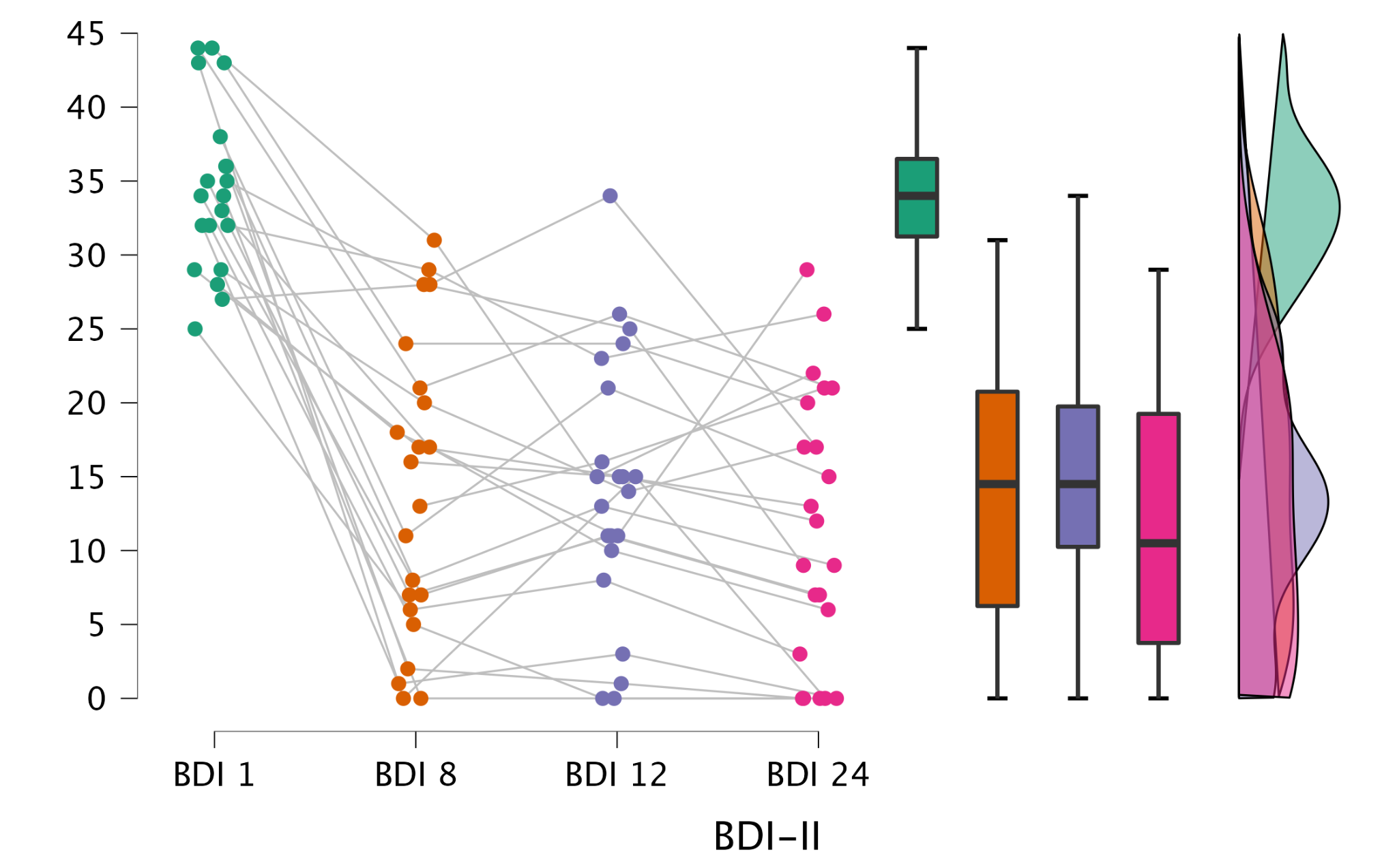

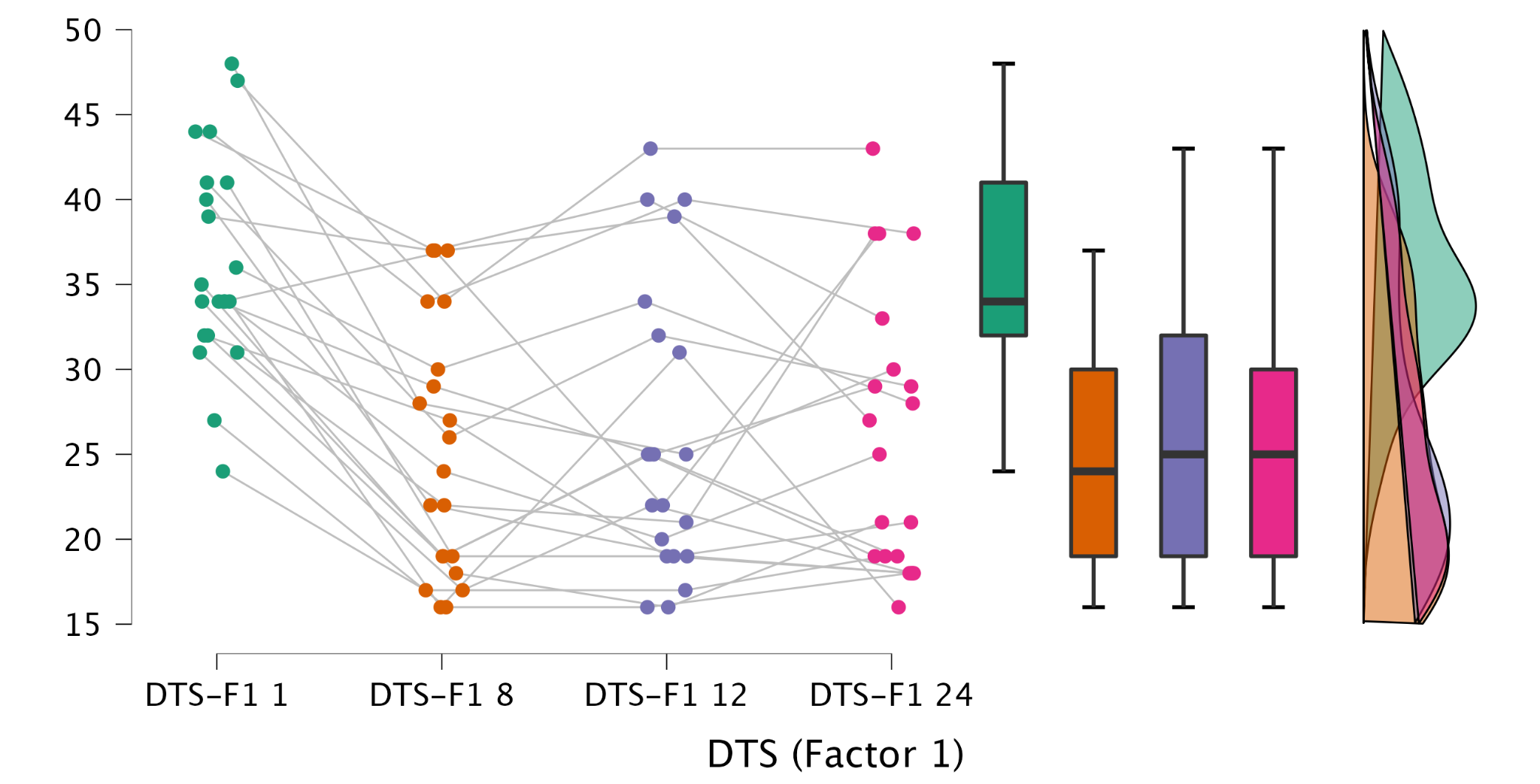


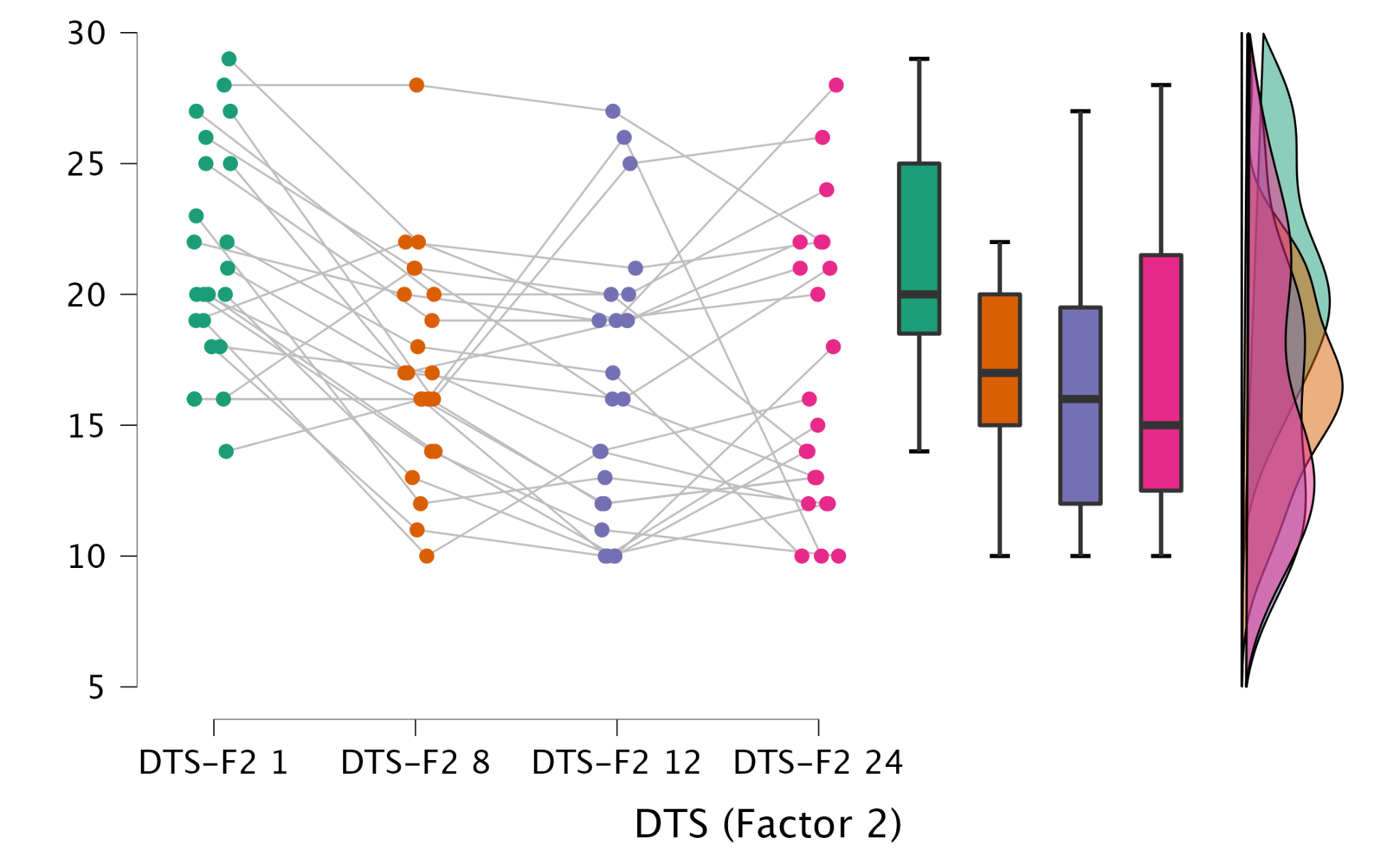


***Note:*** *HAMD = Hamilton Scale of Depression; MADRS = Montgomery Asberg depression scale; BDI-II = Beck Depression Scale 2nd Edition; DTS = Depressive Thoughts Scale; DTS-F1 = factor 1 (low self-esteem/ hopelessness); DTS-F2 = factor 2 (interpersonal relationship).*

**Figure S1**. Analysis of variance of repeated measures (ANOVArm) to compare mean differences longitudinally.
